# Developing an instrument for an early prediction model of long-term functional outcomes in people with acquired injuries of the central nervous system: protocol and methodological aspects

**DOI:** 10.1007/s10072-020-04821-8

**Published:** 2020-10-19

**Authors:** Stefano Masiero, Humberto Antonio Cerrel Bazo, Marcello Rattazzi, Laura Bernardi, Marina Munari, Elisabetta Faggin, Manuela Cattelan, Paolo Pauletto, Alessandra Del Felice

**Affiliations:** 1 Department of Neuroscience, Section of Rehabilitation & NEUROMOVE-Rehab Lab, University of Padova, Via Giustiniani n. 3, 35128 Padova, Italy; 2 Padova Neuroscience Center, University of Padova, Padova, Italy; 3 ORAS (Ospedale Riabilitativo di Alta Specializzazione) Rehabilitation Hospital, Motta di Livenza, Treviso, Italy; 4 Department of Medicine –DIMED, University of Padova, Padova, Italy; 5 Department of Medicine - DIMED, Medicina Generale I^, Ca’ Foncello Hospital, University of Padova, Treviso, Italy; 6 Department of Medicine - DIMED, Institute of Anesthesiology and Intensive Care - Neurointensive Care Unit, Padova University Hospital, Padova, Italy; 7 Department of Statistical Sciences, University of Padova, Padova, Italy; 8 ORAS (Ospedale Riabilitativo di Alta Specializzazione) Rehabilitation Hospital, Motta di Livenza, Treviso, Italy

**Keywords:** Coma, Vegetative state, Minimally consciousness state, Disorders of consciousness, Prognosis

## Abstract

Severe acquired brain injury (ABI) is a major cause of long-term disability and is the main determinant of health and societal costs. Early identification of favourable long-term recovery would allow personalized rehabilitative programs and better health care resources allocation. In light of the higher survival rate from intensive care units (ICU) in recent years, there is a growing need for early prognostication markers of functional recovery; to date, these data have been mainly collected at rehabilitation unit admission and not during the acute phase. We present the protocol and methodology to develop prediction models in people with severe acquired brain injury (GCS at admission to ICU < 8) for the functional and cognitive outcome at 12 months from the event. Predictors will be collected during the acute stage. Participants will be recruited within the first 72 h from the event in the ICUs of two teaching hospitals (Padova and Treviso). Participants will be followed up at discharge from ICU, admission and discharge from Neurorehabilitation and after 12 months from the event. Clinical and functional scales, electroencephalography, evoked potentials, magnetic resonance imaging and serological markers will be entered into a digital registry. Survival will be estimated using the Cox proportional hazard model. A multivariate prediction model will be developed for each of the functional and cognitive outcomes at 12 months from the event.

**Electronic supplementary material:**

The online version of this article (10.1007/s10072-020-04821-8) contains supplementary material, which is available to authorized users.

## Introduction

Severe acquired brain injury (ABI) is a major cause of long-term disability and one of the main determinants of health care and societal costs in terms of DALYs (disability-adjusted life years).

Short-term outcome/survival has mainly been investigated in intensive care units (ICUs) but long-term functional outcomes [1, 2], including social, vocational and cognitive outcomes, have been largely neglected. Yet, the problem is of great clinical and societal importance: early identification of poor recoveries would allow personalized rehabilitative programs and a better allocation of health care resources. It would also support families and caregivers in the emotionally demanding process of acceptance of the outcome of their loved ones.

Functional recovery differs from survival, which is one of the most assessed outcome measures in ICU, as it considers more nuanced aspects of recovery and evaluates the eventual return of the affected person to her/his environment. Long-term care needs, which demands discussion as early as possible with care providers, rely on this prognosis [3].

To date, prognostic determinants of functional outcome have been mainly collected at rehabilitation unit admission, which varies from 1 week to a couple of months from the event, with an average of 38 days [2, 4] or retrospectively, based on clinical scales [5, 6]. These studies included different aetiologies, as is the case in real-world neurorehabilitation facilities, and were based mainly on clinical data. Other long-term studies collected only clinical scores (i.e. Glasgow Coma Scale, quality of life questionnaires or ad hoc questionnaires [7, 8] in traumatic brain injury (TBI) or subarachnoid haemorrhage comatose/post-comatose subjects [5]). Large clinical prospective studies [9, 10] focused on clinical and instrumental (i.e. neuroradiological) prognostic factors for mortality after TBI, but the functional outcome was not an outcome measure and neurophysiological or serological markers were not factored in.

Only recently has the need for specific biomarkers, which could assist in defining long-term functional prognosis [2, 4, 11–13] rather than just survival, being highlighted.

We demonstrated the utility of combining clinical and functional scales with medium latency SSEPs or cortical excitability [14, 15] in the first 72 h from the event in a small sample of post-anoxic comatose persons and people with ischemic stroke to predict the functional outcome at 12 months.

There is a crucial need for more extensive studies in this population to ensure adequate rehabilitative pathways.

We aim to develop a protocol for a multimodal, long-term, functional prognostication model for people with altered consciousness after ABI including neurophysiological, neuroimaging, serological and clinical markers as seen in the early post-injury stages.

### Objectives

#### Primary objectives

To develop prediction models for functional and cognitive outcomes at 12 months from the event in people with severe acquired brain injury (GCS at admission in ICU < 8). Predictors will be clinical, neurophysiological and serological biomarkers collected during the acute phase (< 72 h).

#### Secondary objectives

(i) To evaluate the functional and cognitive recovery trajectories of individuals with severe ABI during the 12 months after ICU admission.
(ii) To investigate differences in the recovery curves among enrolled subjects.

In a subsample (Padova), structural MRI data will be collected and separately analysed. Serum samples will be bio-banked for future studies of correlation with genetic markers.

## Method and analysis

This is a prospective longitudinal registry study, ascertaining predictors of functional and cognitive outcomes. We will report the study protocol using the Transparent Reporting of a multivariable prediction model for the Individual Prognosis Or Diagnosis (TRIPOD) statement for prediction studies. The TRIPOD statement provides recommendations for the reporting of studies developing, validating or updating a prediction model study and consists of a 22-item checklist detailing the essential information that should be included in a report of a prediction model study. It aims at ensuring standardized, high-quality scientific work.

No formal patients and public involvement process were conducted at this stage but a number of informal consultations with patient carers and families were performed to ascertain their wishes. We are now planning to involve the patient associations formally in the process to seek corrections and to help ensure a smooth follow-up.

### Participants

They will be recruited within the first 72 h of severe TBI, intracranial haemorrhage (ICH) and subarachnoid haemorrhage (SAH) in the Intensive Care Units of the University Hospitals in Padova and Treviso, Italy. After discharge, follow-up will be at the Neurorehabilitation Department of Treviso Hospital or at the Rehabilitation Hospital of Motta di Livenza (ORAS). In our settings, subjects with post-anoxic coma are usually admitted to Cardiology ICU and will thus not be included.

Inclusion criteria are a: age 18–80 years; GCS < 8; aetiology: trauma, vascular event, tumour or infectious disease; need for mechanical ventilation.

Exclusion criteria are: severe pre-existing cognitive impairment diagnosed by a specialist and/or previous mini-mental state examination < 23, and drug intoxication.

All subjects will be managed according to the usual standard of care. Biomarkers are part of the standard assessment procedures. Data will be further analysed and published only after obtaining informed consent from a legally authorized representative at any time or from the subject after recovery.

Stratification of participants will be according to the cause of injury (trauma, vascular event, tumour or infectious disease). For sufficiently large groups, prediction models will be fitted to each subgroup to determine whether prognostic factors are different for groups with different injury causes.

The study was approved by the Ethics Committee of the University Hospital of Padova (395/AO/16) and Treviso (236/CE AULSS 9).

### Source of data

This is a prospective longitudinal registry study. Data will be sourced from a digital, tertiary centre-based registry setup in January 2018 encompassing all ABI referred to the involved ICUs with a catchment area of 1.4-m inhabitants in north-east Italy. Enrolment will finish in December 2021 and follow-up in December 2022. Data will be anonymized and researchers will only have access to data from their own sites. The study coordinator will have full access to data.

### Outcome

The primary objective of the study is the development of a prediction model for functional and cognitive outcomes at 12 months from the event.

Among measures that cross the International Classification of Functioning [16], disability and health (ICF) domains, Rancho Los Amigos Level of Cognitive Functioning Scale (Rancho or LCFS) and the Disability Rating Scale (DRS) [17, 18] will be administered and provide primary outcome measures. The LCFS is used due to its simplicity; it correlates with DRS items. LCFS scores at the start of rehabilitation are related to vocational outcomes [19]. DRS is a more versatile outcome measure, tracking an individual from coma to community and may discriminate vocational outcomes based on diverse ABI aetiologies [20]. DRS has also a demonstrated correlation with Glasgow Outcome Scale (GOS) and Functional Independence Measure (FIM) [21]. The DRS was introduced to overcome the poor precision of the Glasgow Outcome Scale (GOS) [22]. Its main strength rests in the capability of measuring general functional changes throughout the course of recovery. The Coma Recovery Scale-Revised (CRS-R) will also be administered: it is unique as it expressly incorporates current diagnostic criteria for coma, vegetative state and the minimally conscious state [23]. CRS-R modifications correlate with the functional outcome at 1 year [24].

Diagnosis according to CRS-R relates to levels of functional disability on the DRS and aids long-term treatment planning.

LCFS, DRS and CRS-R will be administered at the time point in which the subject opens eyes and/or discharge from ICUs, rehabilitative unit and at 12-month follow-up.

Modified Barthel Index (BI), Glasgow Outcome Scale Extended (GCS-E) and Supervision Rating Scale (SRS) at 12 months will be administered and collected as secondary outcomes at admission and discharge from the rehabilitative unit and at follow-up (in person or by phone contact) at 12 months. The outcome definition and measurement method will be the same for all participants.

The BI assesses the ability of self-care by individuals with neuromuscular or musculoskeletal disorders [25]. A limitation is that it looks exclusively at physical impairment and neglects the psychological aspects, which play a key role in ABI [26]. In 1983, the 18 items Functional Independence Measure (FIM) was developed because BI was considered too restricted [27]. In fact, it is still widely used as a basic independence measure administered by paramedics and will thus be included.

The GCS-E [28, 29] is an expanded version of the Glasgow Coma Scale [18], subdividing the upper three categories of the GOS. It classifies global outcome in TBI and is used mainly for research purposes in group comparisons.

The Supervision Rating (SRS) measures the level of supervision that a patient/subject receives from caregivers. The SRS rates level of supervision on a 13-point ordinal scale that can optionally be grouped into five ranked categories [30].

### Predictors

Demographic data will be collected. Routine neurophysiological data obtained in the first 72 h will be recorded. They include EEG scored according to the American Clinical Neurophysiology Society Critical Care statement [31] (see Appendix 1), presence and symmetry of standard SSEP and of medium latency SSEP [32]. In the subsample referred to the Padova ICU, structural MRI data acquired during the first week will be included, specifically T-1-weighted images, diffusion-weighted imaging (DWI) and diffusion tensor imaging (DTI).

Serum samples collected during the first 72 h and at 15 days will be analysed to quantify modifications in the circulating levels of biomarkers linked to the inflammatory response, neuronal injury, and cortical integrity.

The degree of the systemic inflammatory response and its modification over time will be investigated through measurement of serum levels of interleukin-1 beta (IL-1beta), IL-6, IL-18, tumour necrosis factor-alpha (TNF-alpha), high sensitivity C-reactive protein and neutrophil gelatinase-associated lipocalin (NGAL).

A recent development in ultrasensitive techniques also allows the robust quantification in serum and plasma samples of neuronal targets associated with brain injury. In this case, we will look at modification in the circulating levels of major proteins localized in astrocytes and neurons such as Astroglial S100β, glial fibrillary acidic protein (GFAP) and neuron-specific enolase (NSE).

Total tau (T-tau) proteins are known to play a role in the stability of axonal microtubules, while phosphorylated neurofilament heavy chain (pNF-H) is an excellent biomarker of ongoing chronic and acute axonal loss. Both these mediators could represent a useful tool to have an indirect estimation of the degree of axonal damage.

Moreover, we will quantify changes in other neurotrophic factors such as the brain-derived neurotrophic factor (BDNF) which is a promoter of proliferation, survival and differentiation of neurons in the peripheral and central nervous systems. Previous studies also showed that the blood level of this protein reflects ongoing neuronal plasticity and is associated with cortical integrity.

All the determination will be performed through the use of dedicated ELISA kits.

### Sample size

The determining explanatory variables in the prediction models for the functional outcomes scales and for the cognitive outcome at 12 months will be selected with a parametric regression method according to the [33] Lasso procedure based on the penalization of the log likelihood. In this case, power and sample size calculations require not only assumptions about the regressors and the effect size but also an evaluation of what the penalization parameter might be. This typically cannot be done a priori, and no formulae are available for sample size computation when this method is applied.

The Lasso methodology is, however, used as it allows the inclusion of many variables in the model and can also be applied when the number of variables exceeds the number of observations and usual regression models cannot be employed.

### Missing data

Missing data are common in large longitudinal studies and we will deal with this by determining the causes of missingness. If observations are missing at random, complete case analysis will be performed as the estimates remain unbiased.

### Statistical analysis method

Initially, explorative data analysis will be performed and the distributions of variables will be described by their means and standards errors or proportions, depending on the variable.

Survival of participants with severe acquired brain injury in the 12 months after ICU admission will be assessed using the Cox proportional hazard model. To evaluate the effect of many predictors on survival, shrinkage methods will be employed [33].

A multivariate prediction model will be developed for each of the functional outcome scales and for the cognitive outcome at 12 months. As outcomes are measured on ordinal scales, a continuation ratio model [34] will be fitted to the data to evaluate the effect of recorded covariates on the outcomes. Independent variables will be considered the same scores at entry and all the predictors collected, including demographic covariates and data obtained from neuroimaging and neurophysiology. To reduce both the bias and the variance in these models, the determining explanatory variables will be selected with a parametric regression method according to the Lasso procedure based on the penalization of the log likelihood [35]. The final model will be selected on the basis of the Bayesian information criterion (BIC) [36] to identify the most appropriate set of covariates for each score.

Statistical analysis will be performed in R (R Core Team, 2019) [37].

### Risk groups

Risk groups (e.g. ‘high risk’, ‘moderate risk’, ‘low risk’) may make models more accessible, but we will not use this to stratify groups as there is no clear consensus on how to create risk groups or how many groups to use [38]. There are also concerns that the use of risk groups may not be in the best interest of affected individuals [38]

## Reporting

### Participants

The flow of participants through the study, including the number of participants with and without the outcome, and a summary of the follow-up time will be described in the final publication. The characteristics of the participants, including the number of participants with missing data for predictors and outcome, will be provided.

### Model development

The number of participants in each analysis will be provided, and if prediction models will be fitted to participants divided according to the cause of injury, the dimension of each subgroup will be reported. The association between the functional scales or the cognitive scale outcomes and each candidate predictor will be assessed using Fisher’s exact test or the chi-square test, depending on the size of the sample. The simultaneous effect of many variables on the outcomes will be analysed by means of a continuation ratio model.

### Model specification

The continuation ratio model is the appropriate statistical model for the analysis of the functional and cognitive outcomes since these are ordinal categorical variables. The use of penalized regression methods will allow the inclusion of a large number of predictors in the model. For each of the prediction models fitted to the functional and cognitive outcomes, regression coefficients and model intercept will be reported, so that predictions for individuals could be obtained. These models will allow us to compute the probability of gaining a specific level of the functional or cognitive outcome at 12 months for an individual with a specific set of covariates at ICU admission. Examples will illustrate how to compute such probabilities.

Parameter estimates of the Cox survival model will be provided, too.

### Model performance

The evaluation of the performance of the prediction models for the functional and cognitive scales will be based on the quality of the predictions provided. Since an external validation set will not be available, models will be assessed using in-sample predictions that will be compared to actual outcomes by means of the rank probability score [39]. Further performance measures based on accuracy indices, as total error rates and sensitivity and specificity for each level of the scales, will be computed.

### Limitation of the protocol

The main limitation is the lack of inclusion of subjects with post-anoxic coma.

## Conclusion

We have described the methods and statistical analysis plan to develop a prognostication model for functional long-term outcome after acquired brain injury in an adult population. This tool will be one of the firsts of its kind in ABI to follow, a priori, the TRIPOD reporting guidelines for prognostic research. The study predicts outcomes longitudinally, which may be more adequate to clinical needs than predictions made at predefined time points.

Results coming from this study will be interpreted for both clinical and research purposes.

## Electronic supplementary material

ESM 1 (DOCX 14 kb)

ESM 2 (DOCX 87 kb)

## Data Availability

Data from the digital registry will be accessible at any time to identifiable researchers (ORCID number) whose identity may be published only for scientific and non-profit use.
