# Supplementary material for: Developing an instrument for an early prediction model of long-term functional outcomes in people with acquired injuries of the central nervous system: protocol and methodological aspects
Source: Neurol Sci. 2020 Oct 19;42(6):2441–6. doi: 10.1007/s10072-020-04821-8 (PMC8159777; doi:10.1007/s10072-020-04821-8)
Supplement: Supplementary file 1 — (DOCX 14 kb) [file 10072_2020_4821_MOESM1_ESM.docx]

Appendix 1:

Neurophysiological Examination: EEG scoring criteria adapted from Hirsh et al., 2013.

**Background EEG**

| **Simmetry** | □ Simmetric  □ Mild asmmetry (consistent asimmetry in amplitude on referential recording of <50%)  □ Marked asimmetry (differenza interemisferica > 50%) | | |
| --- | --- | --- | --- |
| **Breach Effect:** | □ yes  □ no | | |
| **Predominant background EEG frequency:** | □ α  □ β  □ δ  □ θ | | |
| **Antero-posterior Gradient:** | □ present  □ absent  □ reverse | | |
| **Variability:** | □ yes  □ no  □ unknown/ not applicable | | |
| **Reactivity:** | □ yes  □ no  □ unknown/ not applicable | | |
| **Voltage:** | □ normal  □ low (<20 microV in longitudinal bipolar)  □ suppressed (< 10 microV) | | |
| **Sleep Transients (k-complex and spindles):** | □ normal  □ present but abnormal  □ absent | | |
| **Continuity:** | □ continuous  □ nearly continuous: occasional periods (<10% of the record) of attenuation or suppression  □ discontinuous (attenuation or suppression of 10-49% of the record)  □ burst-attenuation/suppression (more than 50% of the record consisting of attenuation or suppresion) | | |
|  | | |  |
| **RHYTHMIC OR PERIODIC PATTERNS:** | | □ yes  □ no |  |
| **If present** | | □ generalized  □ lateralized  □ bilateral indipendent  □ multifocal |  |
| **Type** | | □ periodic Discharges (PDs)  □ Rhythmic delta activity (RDA)  □ spike Waves (SW) |  |
| **Prevalence** | | □ continuous (>90% of record)  □ abundant (50-89%)  □ frequent (10-49%)  □ occasional (1-9%)  □ rare (<1%) |  |
| **Duration** | | □ very long (>1 h)  □ long (5-59 min)  □ intermediate duration (1-4.9 min)  □ brief (10-59 sec)  □ very brief (<10 sec) |  |
| **Frequency** | | □ <0.5/sec  □ 0.5/sec  □ 1/sec  □ 1.5/sec  □ 2/sec  □ 2.5/sec  □ 3/sec  □ 3.5/sec  □ >4/sec |  |
| **Number of Phases** | | □ 1  □ 2  □ 3  □ >3 |  |
| **Sharpness** | | □ Spiky  □ Sharp  □ Sharply contoured  □ Blunt |  |
| **Amplitude** | | □ Very low (<20 microV)  □ low (20-49 microV)  □ medium ( 50-199 microV)  □ high (> 200 microV) |  |
| **Polarity** | | □ positive  □ negative  □ dipole, horizontal/tangential  □ unclear |  |
| **Stimulus** | | □ stimulus-indotto  □ spontaneous  □ unclear |  |
| **evolving** | | □ yes  □ no  □ unclear |  |
| **Fluttuating** | | □ yes  □ no  unclear |  |
| **Sporadic Epileptiform Discharges** | | □ abundant > 1/10sec  □ frequent > 1/min  □ occasional > 1/h  □ rare <1/h |  |
